# Hydrogen Peroxide-Based Fluorometric Assay for Real-Time Monitoring of SAM-Dependent Methyltransferases

**DOI:** 10.3389/fbioe.2018.00146

**Published:** 2018-10-18

**Authors:** M. Kalim Akhtar, Dhanya Vijay, Saima Umbreen, Chris J. McLean, Yizhi Cai, Dominic J. Campopiano, Gary J. Loake

**Affiliations:** ^1^Department of Chemistry, College of Science, United Arab Emirates University, Al Ain, United Arab Emirates; ^2^Institute of Molecular Plant Sciences, School of Biological Sciences, University of Edinburgh, Edinburgh, United Kingdom; ^3^EastChem School of Chemistry, Joseph Black Building, University of Edinburgh, Edinburgh, United Kingdom; ^4^Manchester Institute of Biotechnology, University of Manchester, Manchester, United Kingdom

**Keywords:** biocatalysis, drug screening, high throughput, Amplex Red, methylation

## Abstract

Methylated chemicals are widely used as key intermediates for the syntheses of pharmaceuticals, fragrances, flavors, biofuels and plastics. In nature, the process of methylation is commonly undertaken by a super-family of S-adenosyl methionine-dependent enzymes known as methyltransferases. Herein, we describe a novel high throughput enzyme-coupled assay for determining methyltransferase activites. Adenosylhomocysteine nucleosidase, xanthine oxidase, and horseradish peroxidase enzymes were shown to function in tandem to generate a fluorescence signal in the presence of S-adenosyl-L-homocysteine and Amplex Red (10-acetyl-3,7-dihydroxyphenoxazine). Since S-adenosyl-L-homocysteine is a key by-product of reactions catalyzed by S-adenosyl methionine-dependent methyltransferases, the coupling enzymes were used to assess the activities of *Eco*RI methyltransferase and a salicylic acid methyltransferase from *Clarkia breweri* in the presence of S-adenosyl methionine. For the *Eco*RI methyltransferase, the assay was sensitive enough to allow the monitoring of DNA methylation in the nanomolar range. In the case of the salicylic acid methyltransferase, detectable activity was observed for several substrates including salicylic acid, benzoic acid, 3-hydroxybenzoic acid, and vanillic acid. Additionally, the *de novo* synthesis of the relatively expensive and unstable cosubstrate, S-adenosyl methionine, catalyzed by methionine adenosyltransferase could be incorporated within the assay. Overall, the assay offers an excellent level of sensitivity that permits continuous and reliable monitoring of methyltransferase activities. We anticipate this assay will serve as a useful bioanalytical tool for the rapid screening of S-adenosyl methionine-dependent methyltransferase activities.

## Introduction

Methylated chemicals are widely used as key intermediates for the syntheses of pharmaceuticals, fragrances, flavors, biofuels, and plastics. In nature, methylation is undertaken by a super-family of enzymes known as methyltransferases which act upon a variety of substrates including proteins, histones, DNA, and RNA (Bauerle et al., 2015). Within a biological context, methylation is considered to be a major epigenetic factor in which the behavior or function of a living organism is subtly altered without modification of its underlying genome sequence (Razin and Riggs, 1980; Dominissini et al., 2012; Boriack-Sjodin and Swinger, 2015). Dysfunction of this process has been implicated in numerous human diseases, cancers and developmental disorders (Robertson, 2005; Kowenz-Leutz et al., 2010; Greer and Shi, 2012). Aside from epigenetics, methyltransferases also play an important role in the syntheses of natural product molecules such as sarcosine, melatonin, and adrenaline (Lew et al., 1977; Nyyssölä et al., 2001; Byeon et al., 2016). Thus, methyltransferases lend themselves well, within an industrial setting, for biomanufacturing purposes (Struck et al., 2012).

For the majority of methyltransferases, S-adenosylmethionine (SAM) serves as the principal methyl donor (Takusagawa et al., 1998). Although a number of mechanistic schemes for SAM-dependent enzymes have been unraveled in recent years, the classical reaction mechanism for methyl transfer proceeds via an SN2 nucleophilic substitution (Takusagawa et al., 1998; Bauerle et al., 2015). The electron-withdrawing property of the sulfur atom of SAM renders the adjacent methyl carbon highly electrophilic. The resulting carbanion becomes prone to attack by nucleophiles residing on the substrate molecule such as nitrogen (present within an amine group) or oxygen (present within hydroxyl or carboxylate groups). This leads to the more stable and uncharged S-adenosyl homocysteine (SAH) along with the methylated product, both of which are released from the enzyme. To aid catalysis, the nucleophilicity of the attacking group may be increased by the presence of the amino acid side-chains of the methyltransferase enzyme.

From a kinetic standpoint, methyltransferases can be difficult and cumbersome enzymes to monitor with reported K_cat_ values as low as 3 min^−1^ (Vilkaitis et al., 2010). In the past, they were typically characterized using radiolabelled substrates based on chromatographic techniques (Akamatsu and Law, 1970; Simon et al., 1978; Smith et al., 1992). Though such techniques offer a high level of sensitivity, radiolabelled substrates are expensive to source and can present a health hazard within the workplace. Additionally, the evaluation of methyltransferases is further complicated by the fact that they often accept a broad range of substrates. For example, the carboxyl-specific methyltransferase (SA MTase) from *C. breweri*, previously characterized by Ross et al. (1999) and Zubieta et al. (2003), methylates not only salicylic acid but also other aromatic substrates such as benzoic acid and vanillic acid. With chromatographic techniques, subtle protocol modifications often need to be made and validated before the desired target chemical can be analyzed which increases research time and effort. A major goal therefore has been to develop rapid and convenient assays that offer the sensitivity and the flexibility to evaluate a broad range of methyltransferases with differing substrate specificities.

Numerous assays already exist for SAM-dependent methyltransferases and these have been comprehensively described in several review articles (Wooderchak et al., 2008; Luo, 2012; Li et al., 2017). These include MS-, antibody-, and probe-based, as well as enzyme-coupled, approaches (Hendricks et al., 2004; Salyan et al., 2006; Graves et al., 2008; Deng et al., 2014; Jin et al., 2016; Neelakantan et al., 2017). An enzyme-coupled approach is particularly appealing as it permits continuous monitoring of the target enzyme which greatly facilitates enzyme characterization and chemical screening studies. With the implementation of microplate technology such assays have become widely adopted (Hendricks et al., 2004; Collazo et al., 2005; Wang et al., 2005; Dorgan et al., 2006; Salyan et al., 2006; Hemeon et al., 2011). The first generic assay for the high-throughput evaluation of methyltransferases was demonstrated in 2004 (Hendricks et al., 2004). In this assay, SAH is hydrolyzed to adenine and homocysteine, and the latter by-product quantified using the classical Ellman's reagent for the detection of thiol groups. The sensitivity of thiol detection can be significantly improved with the use of fluorophores (Collazo et al., 2005; Wang et al., 2005). Rather than monitoring homocysteine, Dorgan et al. (2006) instead shifted their attention to the adenine by-product. By hydrolyzing adenine to hypoxanthine, this chemical change can be monitored at 265 nm. Alternatively, adenine can be used to form ATP, via the enzyme activities of adenine phosphoribosyl transferase and pyruvate orthophosphate dikinase, in order to generate a luciferease-dependent luminescence signal, as previously demonstrated by Ibáñez et al. (2010) and Hemeon et al. (2011). Burgos et al. (2017) devised a one step assay by utilizing SAH deaminase to catabolize SAH to S-inosyl-l-homocysteine which can be monitored at 263 nm. Monitoring in the UV region however is problematic due to the absorption of proteins at 280 nm. To overcome this issue, Duchin et al. (2015), shifted the wavelength to 340 nm by coupling MTase activity to NADPH oxidation, catalyzed by glutamate dehydrogenase. All of the aforementioned assays essentially rely on the quantitative detection of the SAM by-product, S-adenosylhomocysteine (SAH). A principal advantage of these aforementioned assays is that they alleviate inhibitory feedback arising from SAH accumulation.

In this study, we describe a novel enzyme-coupled assay for monitoring methyltransferase activity. The assay relies on the generation of hydrogen peroxide which can be quantified with horseradish peroxidase using the molecular probe, Amplex Red. The assay was validated with methyltransferases that are known to accept DNA and small natural products as substrates. A fluorogenic signal is generated during the assay which offers an excellent level of sensitivity compared to chromogenic assays. Furthermore, we show that the *de novo* synthesis of the relatively expensive and unstable cosubstrate, SAM, can be incorporated within the assay.

## Materials and methods

### Strains and plasmids

The gene encoding for *C. breweri* SA MTase (Uniprot accession number: Q9SPV4), in addition to bases encoding for 6 N-terminal histidine residues, was codon-optimized for expression in *E. coli*, synthesized, and sub-cloned into the pET-15b expression vector using *Nco*I and *Bam*HI restriction sites (Genscript, USA). The genes encoding for *E. coli* Mtn (Uniprot accession number:P0AF12) and MetK (Uniprot accession number: P0A817) were amplified using the HotStar HiFidelity Polymerase Kit (Qiagen). *E. coli* BL21(DE3) genome was used as the template in combination with the following primers: 5′- attaatACTAGTATGGCAAAACACCTTTTTACGTCCGAGTCC- 3′ (*metK* forward) and 5′-attaatCTC GAGTTACTTCAGACCGGCAGCATCGCG-3′ (*metK* reverse), 5′-attaatACTAGTATGAAAATCGGCAT CATTGGTGCAATGGAAG-3′ (*mtn* reverse) and 5′-attaatCTCGAGTTAGCCATGTGCAAGTTTCTGCA CCAGTG (*mtn* forward). The amplified products and an in-house pET-28b-derived vector containing an N-terminal His tag-encoding sequence were doubly digested with *Spe*I (NEB) and *Xho*I (NEB). After gel/column purification (Qiagen), the fragments were ligated using T4 DNA ligase (NEB) and transformation carried out with NEB5-alpha competent cells. The mixture was plated overnight at 37°C on kanamycin-supplemented LB/agar plates. Recombinant plasmids were verified by restriction digestion and transferred to BL21(DE3) for protein expression.

### Protein expression and purification

Overnight LB-grown starter cultures of BL21(DE3) were used to inoculate 50 ml cultures of Overnight Express™ Instant TB Medium (Novagen) at 2% (v/v). After overnight incubation (18–24 h, 28°C, 180 rpm), cells were pelleted and resuspended in a lysis buffer containing 50 mM Tris-HCl (pH 7.5), lysozyme (2 mg/ml), and 2% (v/v) hexane, and incubated for 30 min at room temperature with gentle inversion. The insoluble debris was centrifuged (17,000 g, 5 min) and the supernatant applied to His SpinTrap columns (GE Healthcare). The column was washed five times with 0.5 ml 50 mM Tris-HCl (pH 7.5), and the recombinant his-tagged protein eluted with 80 μl 0.4 M imidazole, prepared in 50 mM Tris-HCl buffer (pH 7.5). Imidazole was removed using Zeba™ Desalt Spin Columns and protein recovery was estimated using the Bio-Rad Protein Assay (Biorad). Proteins were analyzed by SDS electrophoresis using 4–20% precast gradient polyacrylamide gels (ThermoFisher) (Laemmli, 1970).

### Evaluation of methyltransferases

For this study, *Eco*RI MTase (NEB) and SA MTase were used to assess methyltransferase activities. To perform the methyltransferase assay, a 100μl reaction typically contained the following assay components: 3.5 μg purified recombinant Mtn, 1 μg XOD (10 units/mg; Sigma), 0.2μg HRP (300 units/mg; Sigma), 0.1 mM Amplex Red (also known as 10-Acetyl-3,7-dihydroxyphenoxazine; Cayman Chemical), 5 mM MgCl_2_, 0.5 mM SAM (32 mM stock; NEB), 1 mM carboxylic acid substrates or ~45 μg (~14 nM) lambda DNA (0.5 mg/ml; ThermoFisher), 1–3 μg SA MTase or 80 units *Eco*RI MTase, and 50 mM Tris-HCl (pH 7.5) or 50 mM potassium phosphate buffer (pH 7.5). For SA MTase, the mixture was additionally supplemented with 1 mM KCl to stimulate activity, as carried out in an early study (Ross et al., 1999). Reactions were initiated either with addition of the substrate or enzyme. Fluorescence output was monitored in 96-well microplates at 590 nm with excitation set at 530 nm using the Tecan M200 Affinity microplate reader. The reactions were incubated at 30°C without shaking for up to 60 min. Control reactions without addition of the purified enzyme were also included to take into account background fluorescence. All assays were performed in triplicates. The K_m_ and V_max_ values of SA MTase for salicylic acid were determined using GraphPad Prism version 7.00 for Windows (GraphPad Software, La Jolla California USA, www.graphpad.com).

### DNA analysis

Methylation of lambda DNA was verified by agarose gel electrophoresis (Lee et al., 2012). Reaction samples containing 1.5 μg lambda DNA were applied to a 0.8% (v/v) agarose gel containing ethidium bromide with TAE (40 mM Tris-acetate, 1 mM EDTA) as the running buffer. DNA bands were visualized by excitation with UV light.

### *De novo* synthesis of SAM

*De novo* SAM was prepared using a reaction mixture containing 25 mM potassium phosphate (pH 7.5), 2 mM methionine (Sigma), 1 mM ATP (Sigma), 5 mM MgCl_2_ (Sigma), and his-tagged recombinant MetK (0.1 mg/ml). The reaction was incubated at 37°C without shaking for up to 1 h. The concentration of SAM (NEB) and SAH (Cayman Chemical) solutions were determined using an extinction coefficient of 15,400 mM^−1^ at 260 nm (Shapiro and Ehninger, 1966)^.^ Formation of SAM was confirmed by HPLC, as described below. For the assessment of methyltransferase activities, an equal volume of the methyltransferase assay mixture described in the above section (without commercial SAM) was added. The reaction was initiated by the addition of *Eco*RI MTase and SA MTase.

### HPLC analysis

Sample analysis was carried out at room temperature using a Shimadzu Prominence HPLC fitted with a SPD-20AUV-Vis detector. Samples (10 μl) were injected and passed through a Phenomenex C18 (2) Luna column (250 × 4.6 mm × 5 μm) at a flow rate 1 ml/min. For the separation and dection of methyl salicylate, the run was performed in gradient mode using a mobile phase consisting of solvent A (acetonitrile and 0.1% (v/v) formic acid) and solvent B (0.1% (v/v) formic acid) with the UV-Vis detector set at 300 nm. Upon injection, solvent B was linearly increased from 5% (v/v) to 100% (v/v) from 0 to 6 min and then restored to its initial level at 5% (v/v) from 6 to 7 min. The column was re-equilibrated with 5% (v/v) solvent B for 5 min prior to each injection. For the separation and detection of SAM, the analysis was performed in isocratic mode, using a mobile phase composed of 5% (v/v) methanol and 0.1% (v/v) formic acid, with the UV-Vis detector set at 260 nm. The retention times of the analytes were confirmed with commercial standards. For methyl salicylate and SAM, these were ~10.16 and ~2.47 min, respectively.

## Results

### Enzyme-coupled assay for the detection of SAH

**Figure 1:**
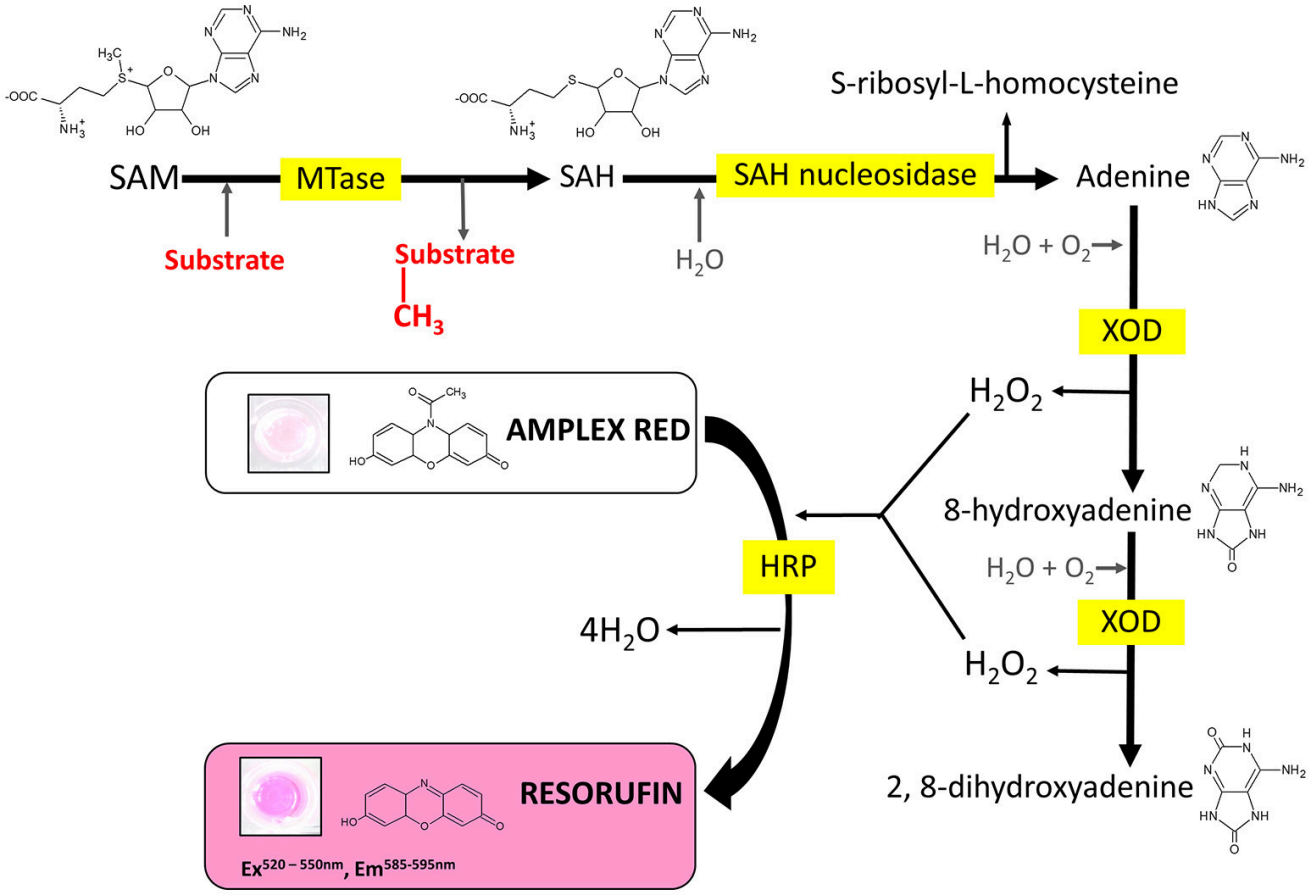
Reaction scheme of the MTase assay. MTase, methyltransferase; SAM, S-adenosyl-L-methionine; SAH, S-adenosyl-L-homocysteine; XOD, xanthine oxidase; HRP, horseradish peroxidase.

**Figure 2:**
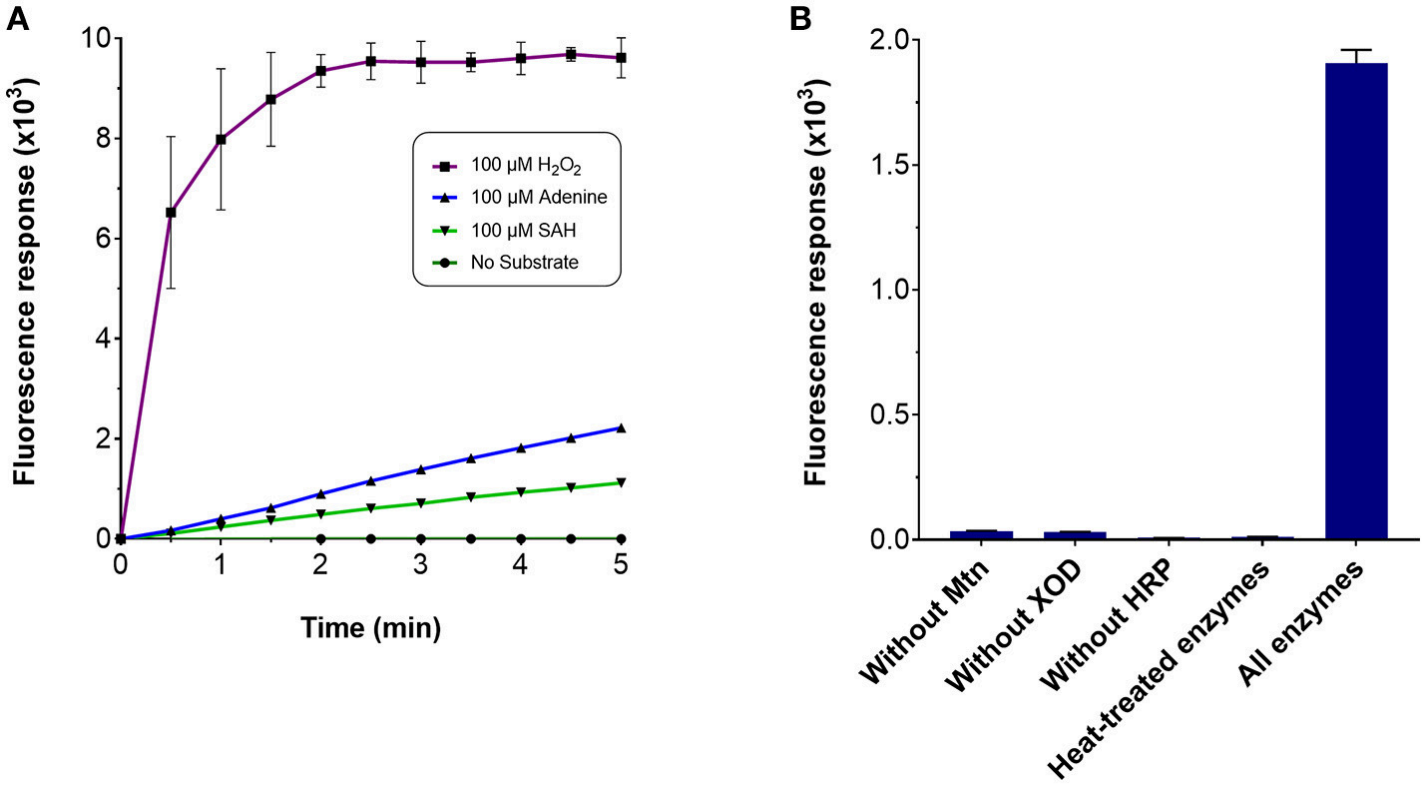
Fluorescence response of the MTase assay. To assess the response of the MTase assay, a 100 μl reaction typically contained the following assay components in 50 mM potassium phosphate buffer (pH 7.5): 3.5 μg purified recombinant Mtn, 1 μg XOD (10 units/mg), 0.2 μg HRP (300 units/mg), and 100 μM of the substrate. Fluorescence response was monitored in 96-well microplates with excitation and emission wavelengths set to 530 and 590 nm, respectively, in the presence of **(A)** 100 μM hydrogen peroxide, 100 μM adenine, 100 μM SAH and in the absence of the aforementioned chemicals, or **(B)** the presence and absence of the assay-coupling enzymes.

Several enzyme-coupled assays have been reported for the quantification of methyltransferase activities (Hendricks et al., 2004; Collazo et al., 2005; Wang et al., 2005; Dorgan et al., 2006; Hemeon et al., 2011). These assays essentially depend on the detection of SAH. Based on this, we considered an alternative enzyme-coupled reaction scheme for monitoring SAH, depicted in Figure 1. Within this scheme, SAH is hydrolyzed by SAH nucleosidase to give homocysteine and adenine, of which the latter could be further oxidized to 2, 8-dihydroxyadenine by xanthine oxidase (XOD) (Wyngaarden and Dunn, 1957). The hydrogen peroxide by-product that is generated can easily be detected in the presence of Amplex Red (10-acetyl-3,7-dihydroxyphenoxazine) and HRP (horseradish peroxidase) which leads to the formation of resorufin, a fluorogenic end-product with excitation and emission wavelengths of 530 and 590 nm, respectively (Kalyanaraman et al., 2012). Since SAH nucleosidase could not be obtained commercially, the gene encoding for *E. coli* Mtn, which possesses SAH nucleosidase activity, was cloned and induced in a T7-based expression system. The recombinant protein was purified via nickel affinity chromatography (Supplementary Figure 1A) (Cornell and Riscoe, 1998). XOD and HRP were both commercially sourced (Sigma, UK). To verify the activity of the coupling enzymes, three substrates: (i) hydrogen peroxide, (ii) adenine, and (iii) SAH were used as the starting points for three separate reactions, with each reaction containing all three enzymes i.e., Mtn, XOD, and HRP. In each case, an increase in fluorescence was observed at 100 μM concentration of hydrogen peroxide, adenine and SAH clearly indicating that the enzymes were fully functional (Figure 2A). The fluorescence increase was found to be dose-dependent from 0 to 100 μM substrate concentration range. The correlation between fluorescence and substrate concentration was highly significant (*r* = 0.961 to 0.981, *N* = 8, *p* < 0.001) and the lines of best fit (*R*^2^ = 0.99) were observed from 0 to 20 μM (Supplementary Figure 2). Further to this, significant fluorescence output was detected only in the presence of active enzymes but not in the presence of heat-treated (i.e., inactivated) enzymes (Figure 2B). Thus, the assay enzymes: Mtn, XOD, and HRP, were able to operate in a tandem manner to generate a fluorescence signal in the presence of SAH, the key by-product of reactions catalyzed by SAM-dependent methyltransferases.

### Continuous monitoring of methyltransferase activity

**Figure 3:**
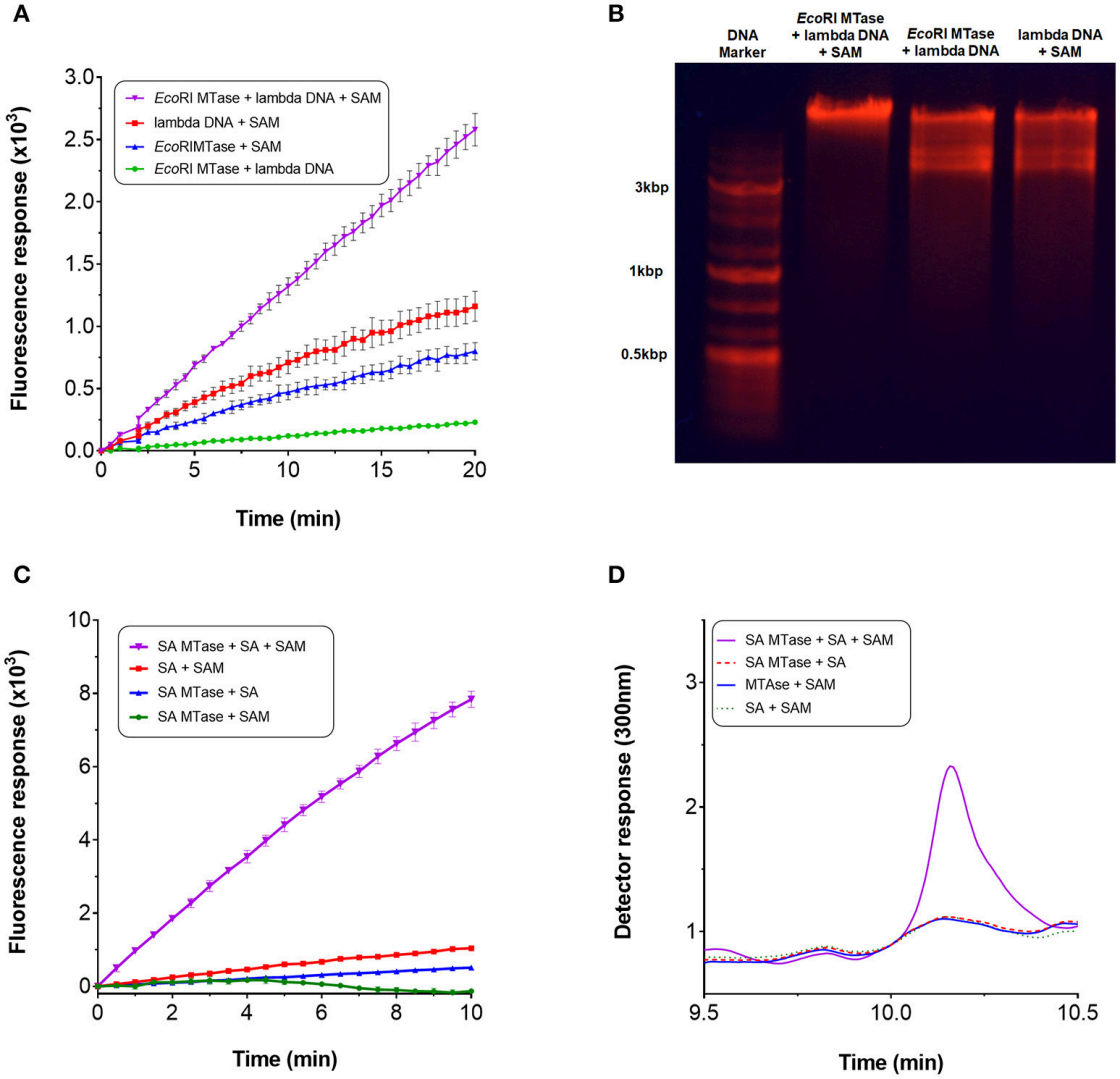
Continuous monitoring of methyltransferases. To perform the methyltransferase assay, a 100 μl reaction typically contained the following assay components: 3.5 μg purified recombinant Mtn, 1 μg XOD (10 units/mg), 0.2 μg HRP (300 units/mg), 0.1 mM Amplex Red, 5 mM MgCl_2_, 0.5 mM SAM (32 mM stock), 1 mM salicylic acid or ~14 nM lambda DNA (0.5 mg/ml), 1-3 μg SA MTase or 80 units *Eco*RI MTase, and 50 mM Tris-HCl (pH 7.5) or 50 mM potassium phosphate buffer (pH 7.5). For SA MTase, the mixture was additionally supplemented with 1 mM KCl to stimulate activity. Reactions were initiated either with addition of the substrate or enzyme. Fluorescence output was monitored in 96-well microplates at 590 nm with excitation set at 530 nm. The reactions were incubated at 30°C without shaking for up to 60 mins. **(A)** Methyltransferase activity of *Eco*RI MTase in the presence of lambda DNA and SAM. **(B)** Confirmation of lambda DNA methylation by agarose gel electrophoresis. **(C)** Methyltransferase activity of SA MTase in the presence of salicylic acid (SA) and SAM. **(D)** Confirmation of the synthesis of methyl salicylate by HPLC.

Next, we qualitatively assessed whether the assay could be used to continuously monitor methyltransferase activity. For this purpose, we employed *Eco*RI MTase which is known to methylate the DNA sequence, GAATTC, of double-stranded linear DNA (Rubin and Modrich, 1977). During the assay, a linear increase in fluorescence intensity was observed upon addition of *Eco*RI MTAse with lambda DNA as substrate at a concentration of 96 nM (Figure 3A). This initial rate of fluorescence increase was found to be at least two-fold greater than the negative controls. The high background fluorescence for the negative controls was most likely attributed to autocatalytic oxidation of the Amplex Red substrate (Summers et al., 2013). The assay was found to be sensitive enough to detect product in the nanomolar range (equivalent to pmol per 100 μl assay volume). Since methylation of the *Eco*RI restriction site imparts resistance against *Eco*RI cleavage activity, the DNA substrate was treated with *Eco*RI to determine whether methylation had taken place. Based on gel analysis, only the methyltransferase-treated DNA in the presence of SAM was found to resist *Eco*RI cleavage indicating that the lambda DNA had indeed been methylated (Figure 3B).

The assay was repeated with another well characterized methyltransferase (SA MTase) from *C. Breweri* which accepts salicylic acid to form methyl salicylate (Chen et al., 2003). A recombinant form of the enzyme was expressed and purified in BL21(DE3) (Supplementary Figure 1B). Again, a strong linear increase in fluorescence was observed with addition of the SA MTase but not in its absence which followed a similar reaction profile as that of *Eco*RI MTase (Figure 3C). The initial rate of fluorescence was found to be at least 60-fold greater compared to the negative controls. The formation of methyl salicylate was confirmed by HPLC (Figure 3D, Supplementary Figure 3). In both cases, fluorescence increase was dependent on the input of SAM clearly demonstrating that the assay can be employed to monitor the activities of SAM-dependent methyltransferases in a continuous manner.

### Characterization of the *C. brewerii* salicylic acid methyltransferase

**Figure 4:**
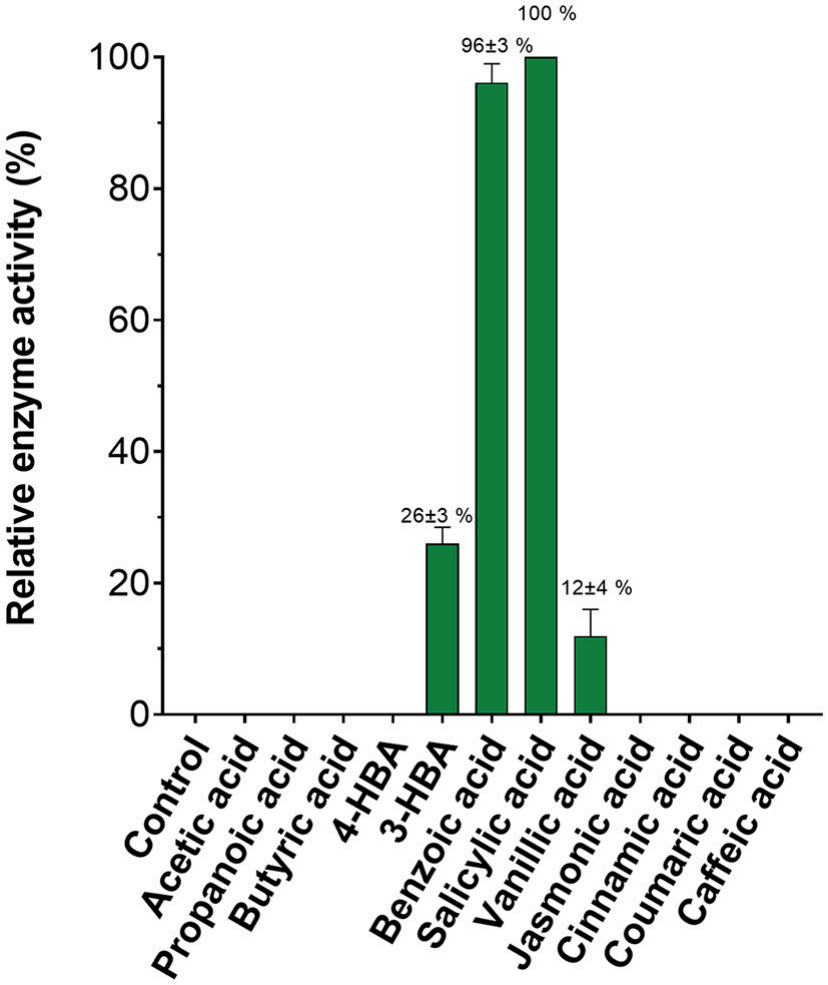
Substrate specificity of *C. Breweri* SA MTase. The reactions were carried out as described in the Figure 3 legend in the presence of aliphatic and aromatic carboxylic acid substrates.

Given the ease with which SA MTase activity could be detected using this assay, we assessed the substrate profile of SA MTase. Zubieta et al. (2003) previously reported that SA MTase accepts a broad range of aliphatic and aromatic carboxylic acids as substrates. Akin to this earlier study, the activity of SA MTase was expressed as a percentage relative to its observed activity with salicylic acid which in this case was determined to be 20.3 nmol product/mg SA MTase/min. Detectable activities were observed only for the aromatic substrates with the order of activity as follows: salicylic acid (100%), benzoic acid (96 ± 3%), vanillic acid (26 ± 3%), and 3-hydroxybenzoic acid (12 ± 4%) (Figure 4). For the remaining substrates, the reaction rates could not be conclusively determined due to the background fluorescence observed within the control reactions.

We also assessed the kinetic characteristics of SA MTase using the currently developed assay. Initial reaction rates were determined using salicylic acid concentrations ranging from 0 to 150 μM (Supplementary Figure 4A). To evaluate whether fluorescence readout correlated well with product formation, we recorded the final fluorescence readings and also determined methyl salicylate (MSA) formation from HPLC analysis (Supplementary Figures 4B,C). The Pearson correlation coefficient analysis revealed that the correlation between fluorescence and methyl salicylate formation (from 0 to 150 μM substrate concentration range) was highly significant (*r* = 0.876, *N* = 19, *p* < 0.001). Excellent linearity (*R*^2^ = 0.94) was noted from 0 to 10 μM of salicylic acid substrate (Supplementary Figure 4D). Based on this linearity, in which 1,000 fluorescence units corresponded to ~9 μM product, methyl salicylate levels as low as 3.6 pmol per 100 μl total reaction volume per well (equivalent to 36 nM of product) could be detected. From this, the rate of fluorescence increase was converted to the rate of product formation, and the apparent K_m_ and V_max_ values of 6.8 (±1.0) μM and 23.8 (±0.5) nmol mg (SA MTase)^−1^ min^−1^ were obtained from non-linear regression analysis (Supplementary Figure 4E). The catalytic turnover was determined to be 0.98 (±0.3) min^−1^.

### Incorporating the *de novo* synthesis of SAM for the methyltransferase assay

**Figure 5:**
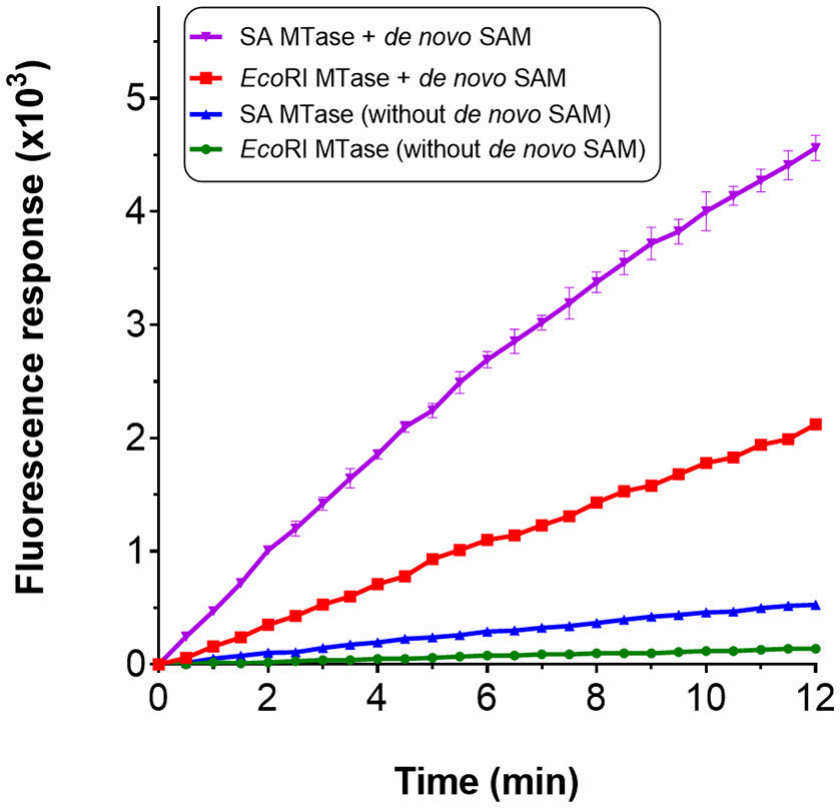
Incorporation of *de novo* SAM for the MTase assay. *De novo* SAM was prepared using a reaction mixture containing 25 mM potassium phosphate (pH 7.5), 2 mM methionine, 1 mM ATP, 5 mM MgCl_2_ and his-tagged recombinant MetK (0.1 mg/ml). The reaction was incubated at 37°C without shaking for up to 1 h. HPLC chromatogram confirming SA MTase in the presence of *de novo* SAM.

We considered the possibility of incorporating an additional step within the assay for the *de novo* synthesis of SAM. Gross et al. (1983) had shown in an early study that it was possible to biologically synthesize SAM *in vitro* from methionine and ATP in the presence of methionine adenosyltransferase. In *E. coli*, this activity is catalyzed by MetK. The gene encoding for this enzyme was therefore cloned and the resulting gene product purified via nickel affinity chromatography (Supplementary Figure 1A). For synthesis of SAM; methionine, ATP, magnesium chloride and MetK, were pre-incubated at 37°C over a 60 min period. An incubation time of 15 min was found to be sufficient to generate ~0.8 mM SAM which corresponded with the retention time of a commercial preparation of SAM (Supplementary Figures 5A,B). Rather than using commercially purified SAM, this additional enzyme-mediated step was incorporated within the assay to serve as the source of SAM. The compatibility of this enzyme step for the assay was tested by using *Eco*RI MTase and SA MTase. For both methyltransferase enzymes, a clear increase in fluorescence was observed with the presence of the *de novo* SAM but not in its absence (Figure 5). The methlyated products were confirmed by gel electrophoresis and HPLC (Supplementary Figures 6A,B). Clearly, the MetK-mediated generation of SAM could be incorporated within the enzyme-coupled assay.

## Discussion

In this study, we have developed an assay for SAM-dependent methyltransferases. The assay was validated using *Eco*RI MTase and SA MTase. By indirectly monitoring SAH, which is a natural by-product of the methyltransferase reaction, the assay allows reliable monitoring of methyltransferase activity. All coupling enzymes were frozen prior to performing the assay indicating that the enzymes were stable enough to withstand at least one freeze/thaw cycle and sufficiently durable for the short time-frame of the assay. The assay, which can be used to complement HPLC and GC-MS analyses, offers two advantages over chromatographic approaches. Firstly, it allows real-time, continuous monitoring of methyltransferase activities. Secondly, it could be applied in high throughput systems for monitoring multiple samples in parallel.

Though there is a wide variety of commercialized methyltransferase assay kits on the market, they are prohibitively expensive for research labs with constrained budgets to implement on a routine basis. Furthermore, the proprietary nature of commercialized kits, which prevents full disclosure of the assay details, makes it inherently difficult to customize the analytical approach should such a need arise. The assay developed within this study has a similar reaction scheme to the commercial methyltransferase assay kit currently available from G-Biosciences, Cayman Chemicals and Merck (sold as “SAM Methyltransferase Assay” or “Methyltransferase Colorimetric Assay Kit”). The commercial assay and the one reported within this study both rely on the generation of hydrogen peroxide for methyltransferase activity detection. However, for the commercialized kit, the detection of hydrogen peroxide is achieved using the colorimetric agent, 3,5-dichloro-2-hydroxybenzenesulfonic acid (DHBS) which has an extinction coefficient 26,000cm^−1^ M^−1^ at 510 nm. For this study, the detection of hydrogen peroxide is made possible with use of resorufin, the oxidized product of Amplex Red. Resorufin has an extinction coefficient of 57,000 cm^−1^ M^−1^ at 570 nm. Moreover, resorufin is a fluorescent end-product with excitation and emission wavelengths of approximately 530 and 590 nm, respectively, and a quantum efficiency of 0.97 (Gomes et al., 2005). From an analytical standpoint, the fluorescence and absorption wavelengths of resorufin are ideal for continuous detection as they lie well away far from the maximum absorption wavelengths of many biological molecules such as NADPH (at 340 nm) and proteins (at 280 nm). Thus, resorufin serves as a superior probe for hydrogen peroxide detection.

We show also that SAM can be very easily generated in-house, as demonstrated previously by Gross et al. (1983), and incorporated within the MTase assay. As a co-substrate, the use of commercially sourced SAM is problematic for two main reasons. Firstly, it is expensive to obtain in a highly purified form; for research purposes the cost of molecular-grade SAM can be as high as ~$5/mg. Secondly, SAM is relatively unstable at physiological pH and prone to degradation (Hoffman, 1986; Desiderio et al., 2005). It can exist in the R/S and S/S enantiomers, of which the former is the biologically relevant enantiomer for methyltransferase activity whereas the S/S form is inhibitory (Quinlan et al., 2013). In light of these issues, SAM is typically stored at acidic pH. Here, we show for the first time that MetK-mediated synthesis of SAM can be used to support methyltransferase activity. Such an approach would cheapen the cost of performing MTase assays, or even SAM-dependent bioconversions, on a large- scale, and reduce issues relating to the long-term storage of SAM.

In this study, the apparent K_m_ and V_cat_ values of 6.8 μM and 0.98 min^−1^ for the SA MTase were considerably less than the previously reported values of 23.0 μM and 5.5 min^−1^ by Zubieta et al. (2003), respectively. The lower kinetic values obtained in this study can be explained by the poor fluorescence response observed with increased salicylic acid synthesis, as confirmed by HPLC. This is most likely due to the kinetic constraints of one or possible more of the coupling enzymes. We suspect that xanthine oxidase may provide insufficient coupling activity for two reasons. Firstly, the standard curve shows substantially weaker fluorescence for adenine and SAH compared with hydrogen peroxide at equimolar amounts (refer to Figure 2A). Secondly, xanthine oxidase has previously been reported to have a K_cat_/K_m_ value of 1.8 × 10^4^ s^−1^ M^−1^ for adenine which can become reduced to 3.8 x × 10^1^ s^−1^ M^−1^ in the presence of high adenine concentrations due to the effects of cooperativity (Tai and Hwang, 2011). In contrast, the SAH nucleosidase and horseradish peroxidase display higher K_cat_/k_m_ values of 2.0 × 10^6^ M^−1^ s^−1^ (Lee et al., 2005) and 2.5 × 10^6^ M^−1^ s^−1^ (Glettenberg and Niemeyer, 2009) for the SAH and Amplex Red substrates, respectively. Given the possible kinetic contraints, one possible solution would be to increase the amount of coupling enzymes used for the assay. Yet, another workaround solution might be to modify the reaction scheme. Adenine deaminase could instead be employed to convert adenine to hypoxanthine; the latter could then be converted by xanthine oxidase to uric acid. Under optimal conditions, adenine deaminase enzyme displays a far more favorable K_cat_/k_m_ value of 5.0 × 10^6^ M^−1^ s^−1^ for adenine without negative cooperative effects (Kamat et al., 2011). Further experimentation would be needed to assess the feasibility of the assay for kinetic purposes. In particular, the kinetic efficacy of each assay step, as well as the stability of the assay enzymes, would be highly insightful for further developing and refining the assay to allow reliable kinetic analyses.

Nonetheless, the overall trend in activity with regard to the substrate profile was found to be in relatively good agreement with the Zubieta et al. work (Zubieta et al., 2003). The assay can therefore still be employed to qualitatively detect methyltransferase activities. In addition to substrate profiling, the assay could also be applied for the screening of novel drugs that inhibit methyltransferases. Recent studies utilizing high throughput methyltransferase assays have led to the evaluation of drug inhibitors such as 5-azacytidine and 5-aza-2′-deoxycytidin (Chen et al., 2018; Foik et al., 2018). In summary, this assay serves as a rapid, real-time monitoring bioanalytical tool for the detection of methyltransferase activities and likely to have useful applications in the syntheses of methylated products for the fuel, agrochemical and pharmaceutical industries.

## Author contributions

MKA, GJL and DJC helped to develop the research concept, and advised on experimental design, data interpretation, and writing the manuscript. MKA conceived the research, designed and carried out experiments. DV composed and formatted the figures. SU assisted in experiments. CJM assisted in data analysis. YC assisted in editing and writing the manuscript.

### Conflict of interest statement

The authors declare that the research was conducted in the absence of any commercial or financial relationships that could be construed as a potential conflict of interest.
