# Supplementary material for: Hydrogen Peroxide-Based Fluorometric Assay for Real-Time Monitoring of SAM-Dependent Methyltransferases
Source: Front Bioeng Biotechnol. 2018 Oct 18;6:146. doi: 10.3389/fbioe.2018.00146 (PMC6200863; doi:10.3389/fbioe.2018.00146)
Supplement: Supplementary file 1 [file Data_Sheet_1.pdf]

Supplementary Figure 1

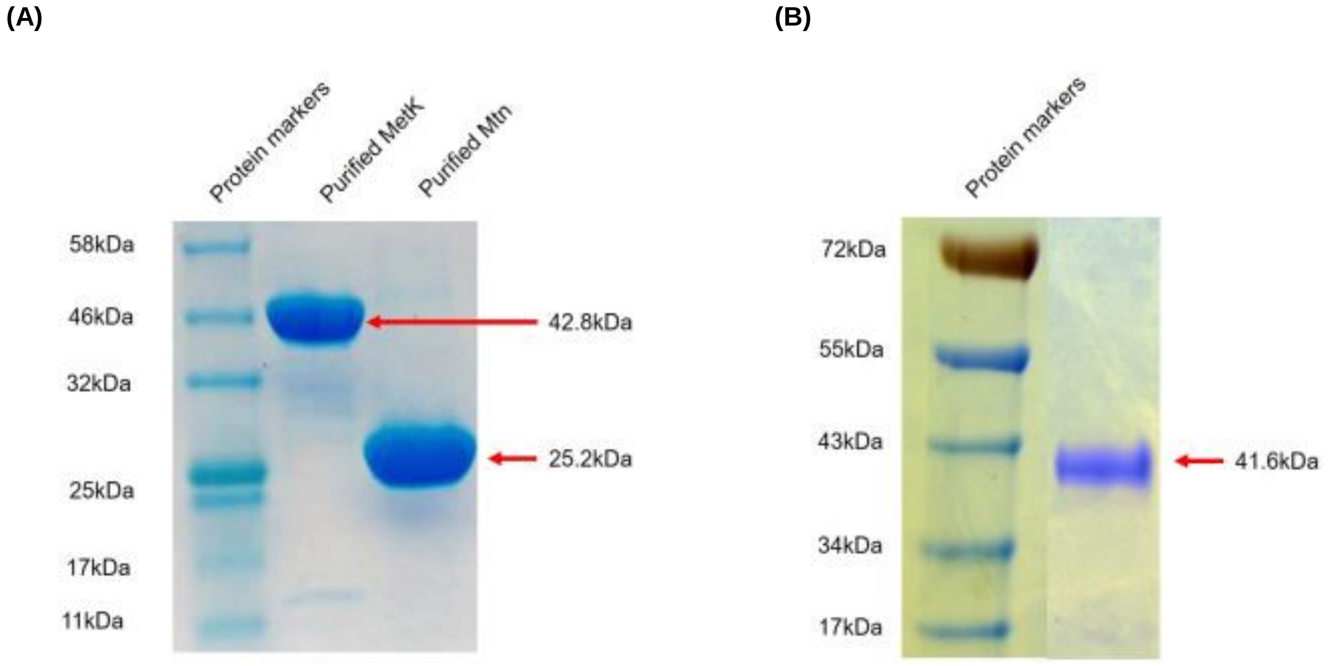

Supplementary Figure1. SDS-PAGE profile of purified protein preparations. (**A**) Purified preparation of MetK and Mtn. (**B**) Purified preparation of SA MTase from *C. breweri.*

Supplementary Figure 2

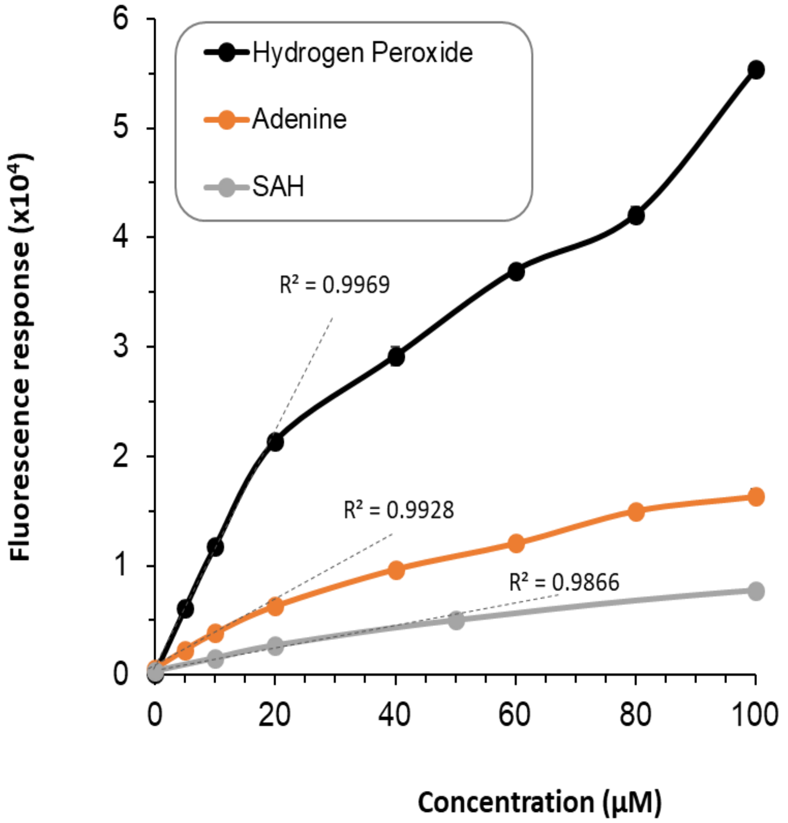

Supplementary Figure 2. Fluorescence response of the MTase assay in the presence of hydrogen peroxide, adenine, and SAH. Four reactions were set up, as described in the material and methods, using three starting substrates: hydrogen peroxide, adenine, and SAH. Substrate concentration ranged from 0 to 100mM. Fluorescence output was monitored with excitation and emission wavelengths set to 530nm and 590nm, respectively. Error bars represent standard deviation (n=3).

Supplementary Figure 3

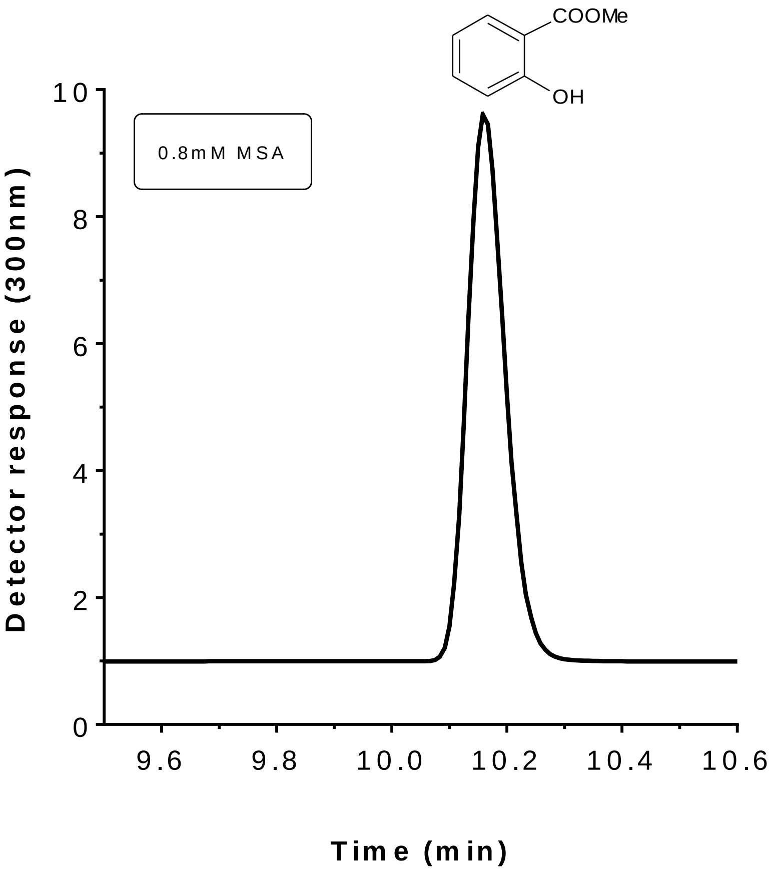

Supplementary Figure 3. HPLC chromatogram of a methyl salicylate (MSA) standard. With the detector response set at 300nm, the retention time was found to be ~10.2 minutes.

Supplementary Figure 4

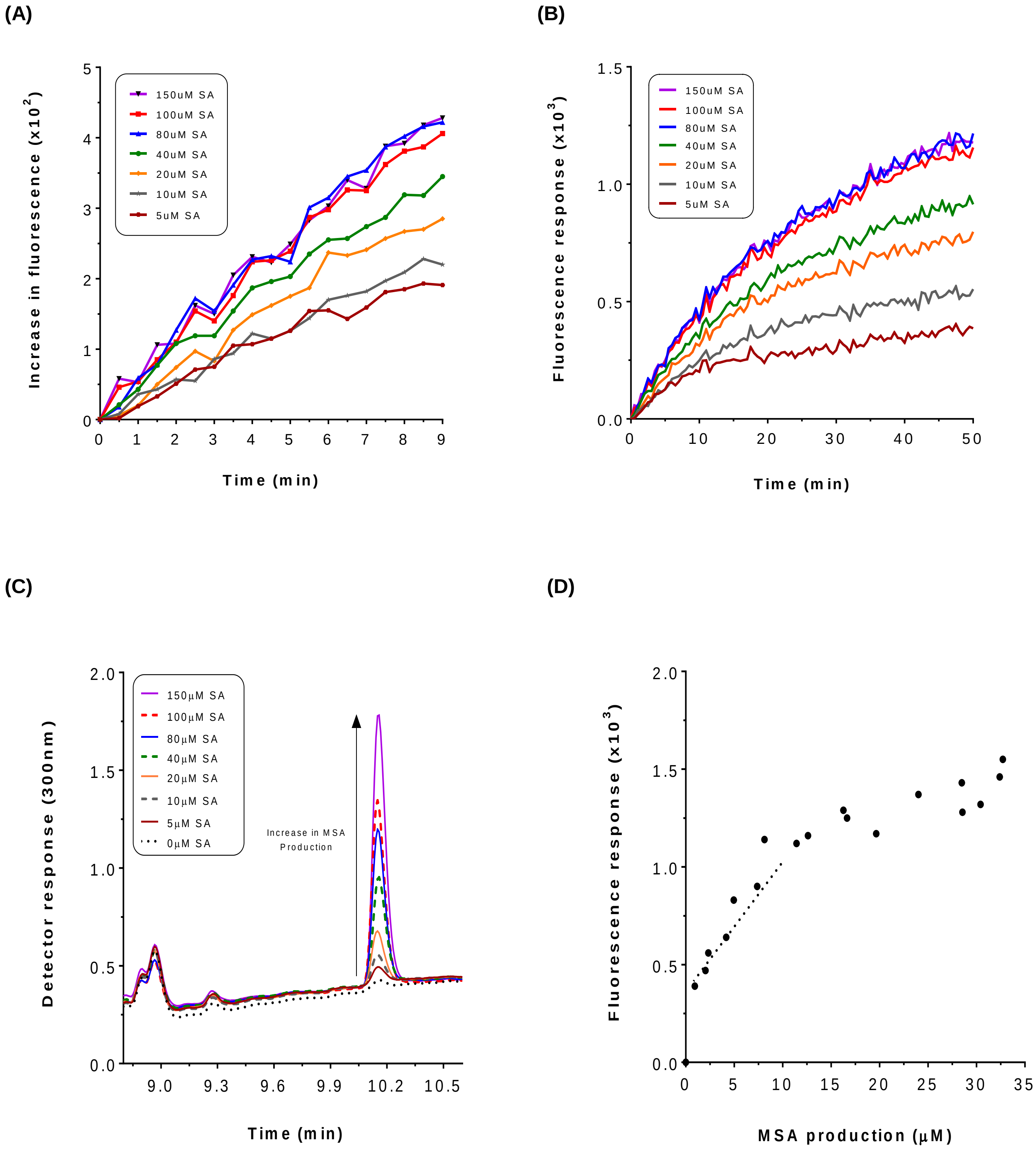

(E)

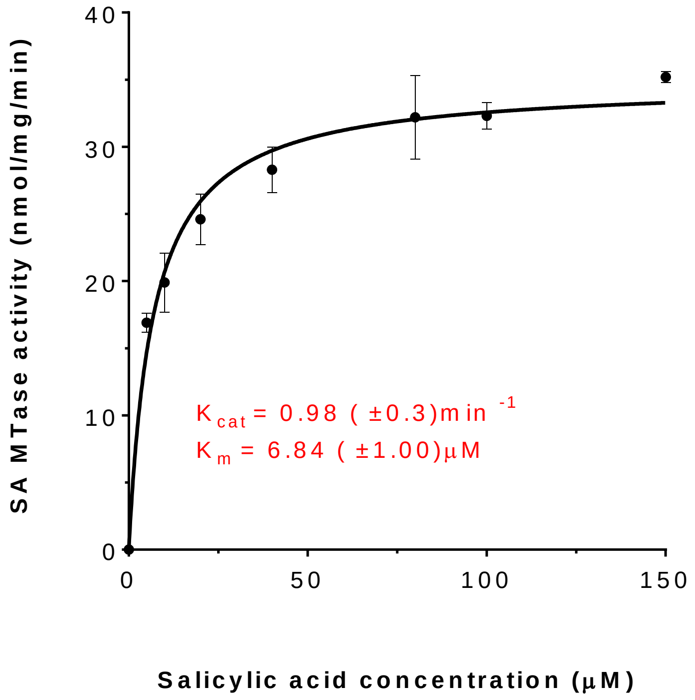

Supplementary Figure 4. Evaluation of fluorescence emission and methyl salicylate production. Reaction conditions were set up as described in the Materials and Methods and carried out in the presence of salicylic acid at a concentration range from 0 to 150 μM. The reactions were monitored via fluorescence emission at 590nm. After removing background fluorescence, the increase in fluorescence was plotted against time to (**A**) determine the initial reaction rates of SA MTase and (**B**) generate a time-course fluorescence profile over the duration of the assay. (**C**) The actual concentration of methyl salicylate (MSA) produced was determined by HPLC analysis. (**D**) A scatter plot of fluorescence emission versus methyl salicylate production was generated. A linear relationship (indicated by the dashed arrow) was used to convert fluorescence emission to methyl salicylate production for the kinetic analysis of SA MTase. **(E)** Kinetic assessment of SA MTAse, determined using the currently developed MTase assay, for salicylic acid (0- 150μM).

Supplementary Figure 5

(A)

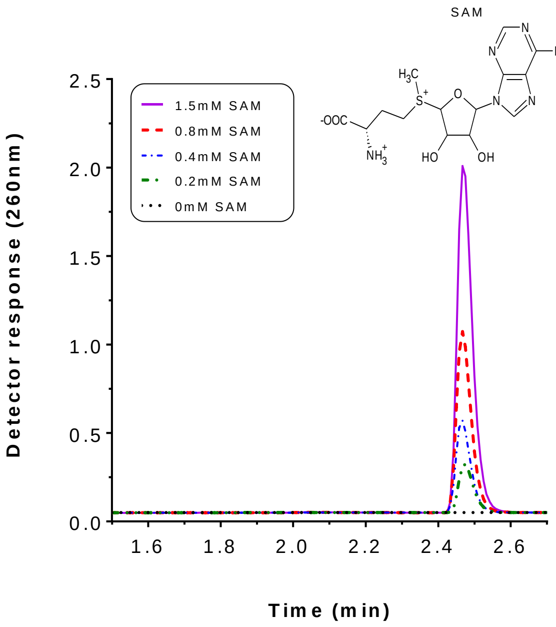

(B)

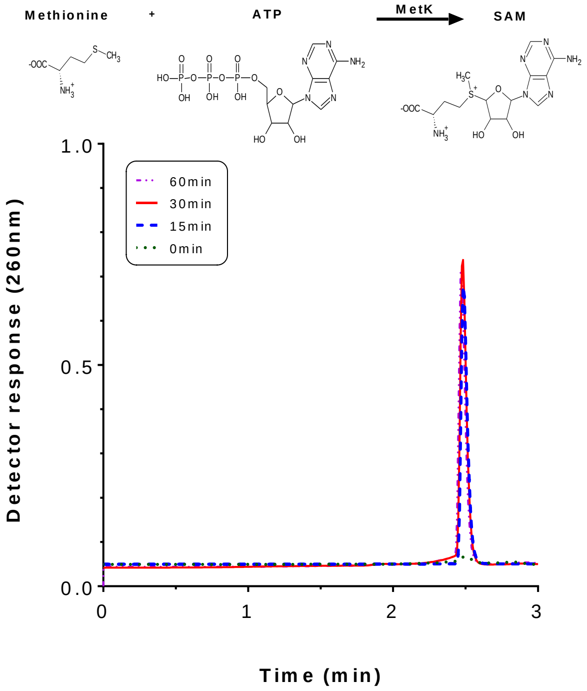

Supplementary Figure 5. *De novo* production of SAM. (**A**) HPLC chromatogram of commercially sourced SAM prepared at 0 to 1.5 mM concentration. The retention time was found to be ~2.47 minutes. Detector response was based on absorption at 260 nm. (**B**) HPLC chromatogram of enzyme-synthesised SAM prepared using a reaction mixture containing 25 mM potassium phosphate (pH 7.5), 2 mM methionine, 1 mM ATP, 5 mM $MgCl_2$ and his-tagged recombinant MetK (0.1 mg/ml). The reaction was incubated at 37°C without shaking for up to 60 min.

Supplementary Figure 6

(A)

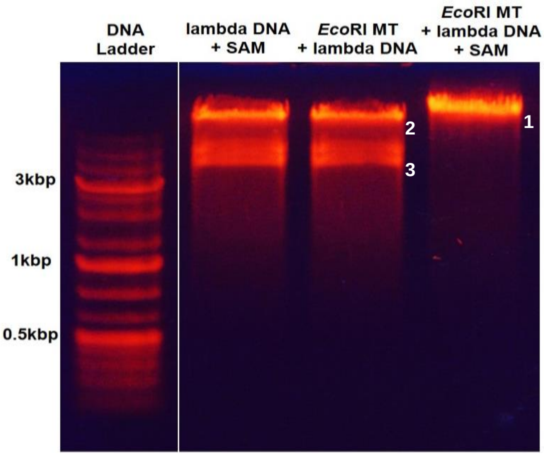

(B)

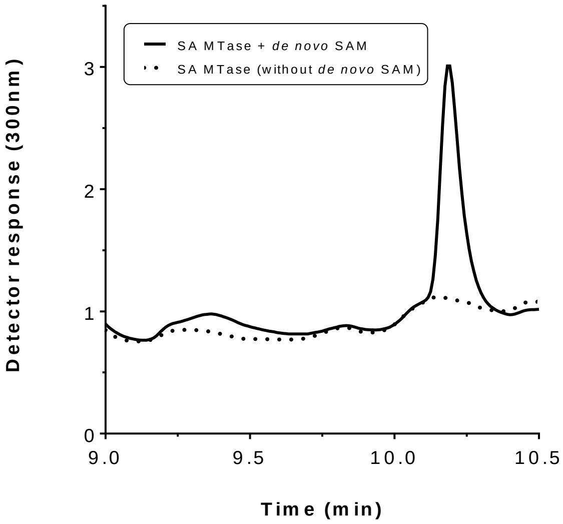

Supplementary Figure 6. Methyltransferase activities of *Eco*RI and SA MTase. (**A**) Agarose gel confirming methylation of lambda DNA. Region 1 corresponds to uncut lambda DNA (48.5kbp). Regions 2 (21.2 kbp) and 3 correspond to the *Eco*RI-cut fragments of lambda DNA: 7.4 kbp, 5.8 kbp, 5.6 kbp, 4.9 kbp, 3.7 and 3.5 kbp. (**B**) HPLC chromatogram showing methyl salicylate which has a retention time of ~10.2 minutes.
